# The Cell Cycle Regulator CCDC6 Is a Key Target of RNA-Binding Protein EWS

**DOI:** 10.1371/journal.pone.0119066

**Published:** 2015-03-09

**Authors:** Sujitha Duggimpudi, Erik Larsson, Schafiq Nabhani, Arndt Borkhardt, Jessica I Hoell

**Affiliations:** 1 Department of Pediatric Oncology, Hematology and Clinical Immunology, Center for Child and Adolescent Health, Heinrich Heine University, Medical Faculty, Duesseldorf, Germany; 2 Department of Medical Biochemistry and Cell biology, Institute of Biomedicine, The Sahlgrenska Academy, University of Gothenburg, Sweden; Tulane University School of Medicine, UNITED STATES

## Abstract

Genetic translocation of EWSR1 to ETS transcription factor coding region is considered as primary cause for Ewing sarcoma. Previous studies focused on the biology of chimeric transcription factors formed due to this translocation. However, the physiological consequences of heterozygous EWSR1 loss in these tumors have largely remained elusive. Previously, we have identified various mRNAs bound to EWS using PAR-CLIP. In this study, we demonstrate CCDC6, a known cell cycle regulator protein, as a novel target regulated by EWS. siRNA mediated down regulation of EWS caused an elevated apoptosis in cells in a CCDC6-dependant manner. This effect was rescued upon re-expression of CCDC6. This study provides evidence for a novel functional link through which wild-type EWS operates in a target-dependant manner in Ewing sarcoma.

## Introduction

**Fig 1:**
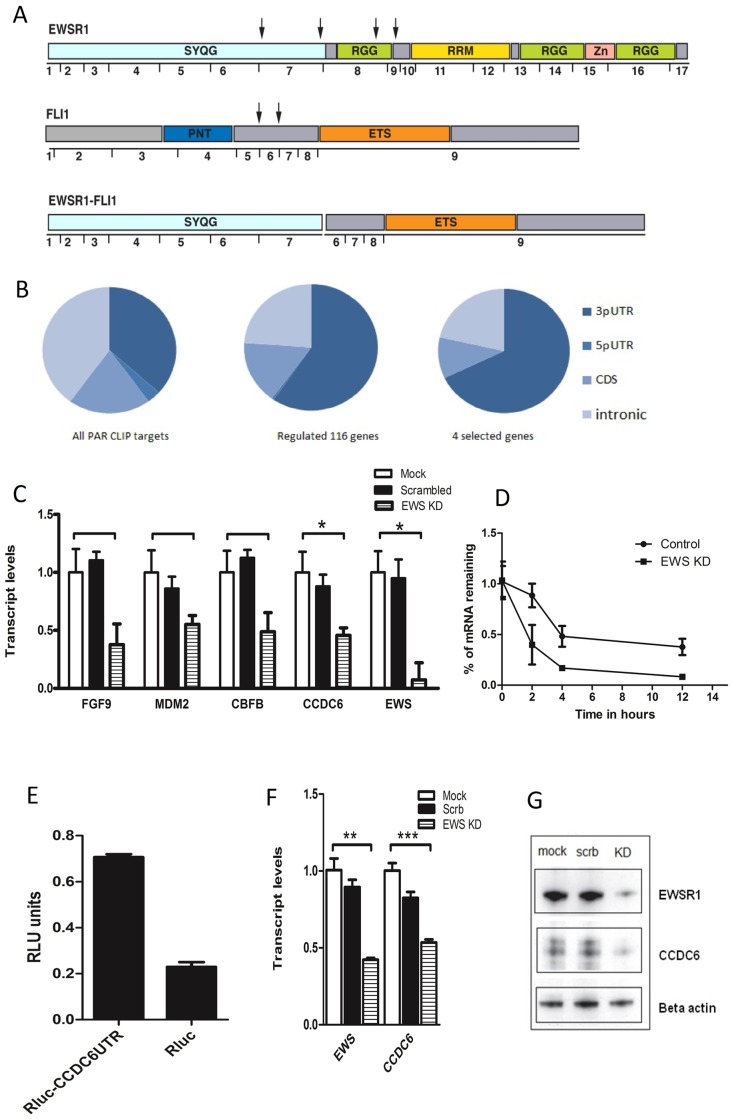
Regulation of targets by EWS in vivo. **A**. Protein domain organization of EWS and FLI1. The black vertical arrows indicate common breakpoints in Ewing sarcoma. Numbers correspond to exons and a typical EWS-FLI1 fusion protein is also shown. Note that the RNA-binding domain of EWS is lost in the process of translocation. **B**. Pie diagram showing the distribution of PAR-CLIP clusters across 3’UTR, 5’UTR, intronic and coding regions of Refseq RNAs. The three diagrams give the cluster distribution of all sequenced EWS PAR-CLIP targets, all targets regulated by EWS and the four targets we validated (FGF9, MDM2, CBFB, CCDC6). **C**. Relative mRNA levels of targets genes FGF9, MDM2, CBFB, CCDC6 and EWS in HEK293T cells following EWS knockdown assayed by qRT-PCR (mock: only transfection reagent used; scrambled: AllStars Negative Control siRNA; EWS: siRNA targeting EWS). Relative mRNA levels were normalized to beta actin and quantified relative to the mock and scrambled control levels. Results are shown as mean SEM values (*P < 0.05; n = 3 per group). **D**. Amount of CCDC6 mRNA transcript percentage is measured upon knocking down of EWS as compared to control. The level of transcript was measured by qRT-PCR after knocking down for 24 hours followed by treatment with actinomycin D. The linear regression and slopes were calculated and the data is presented as Mean and SEM on a linear scale. **E**. Luciferase activity of CCDC6 upon EWS transfection (normalized to empty psiCHECK-2 plasmid). Data is shown as the fold increase in luciferase activity (RLU units) relative to control. Results are shown as the mean SEM values (*P<0.05; n = 3 per group). **F**. Relative mRNA levels of CCDC6 and EWS in mock, control and EWS knockdown in MHH-ES-1 cells. Knockdown of EWS decreased the expression of CCDC6. The mRNA levels were normalized to beta actin. Data is represented as mean SEM values (*P<0.05; n = 3 per group). **G**. Western blot showing the downregulation of CCDC6 upon EWS knockdown in MHH-ES-1 cells. Antibodies are indicated.

Ewing sarcoma which was first reported by James Ewing in 1921 is the second most common bone and soft tissue malignancy in adolescents and young adults [1, 2]. Genetically, 90% of these tumors are characterized by a translocation whereby the N-terminal portion of the RNA-binding protein (RBP) EWSR1 is joined to a DNA-binding protein belonging to the ETS family of transcription factors (e.g. FLI1, ERG, and ETV1) (Fig. 1A). Additionally, EWSR1 fusions to ATF1 cause soft tissue clear cell sarcoma while fusions to CHOP cause myxoid liposarcoma [3, 4]. Considering that the resulting chimeric transcription factors such as EWS-FLI are under the control of the strong FET promoter, they are expressed at high levels in the cell and generally believed to be the main cause of malignant transformation. Additionally, the loss of the EWSR1 allele creates haploinsufficiency of EWS protein which affects its RNA-binding activity and also its mRNA targets suggesting that EWS and its targets have important roles in the development of disease [5]. It has also been shown that EWS/FL1 alone is not sufficient to induce sarcomagenesis in a transgenic mouse model suggesting that factors unrelated to the aberrant transcription factors also contribute to the development of Ewing sarcoma family tumours (ESFT) [6].

EWS is one of roughly 600 RNA-binding proteins (RBPs) which play important roles in mRNA stability, transport and cellular localization [7]. It belongs to the FET family of RBPs which includes FUS, EWS and TAF15. These proteins bind to RNA and DNA and are implicated in the regulation of gene expression and cellular signalling. Altered protein expression of these ubiquitously expressed proteins has been shown to cause various human cancers [3, 8] EWS affects cellular growth mechanism like proliferation, migration and invasion by regulating AKT substrate PRAS40 [5] and FAS dependent apoptosis by regulating the exon skipping of FAS/CD90 [9]. EWS plays a major role in mitosis during spindle formation by regulating Aurora B kinase [10, 11]. EWS knockout mice showed defects in B-lymphocyte development, meiosis, spermatocyte development, interferon signalling and HSC dynamics suggesting an important role of EWS in DNA damage response (DDR) [12]. Together, EWS showcases a promising role in several cell survival pathways.

To get a deeper insight into the physiological function of EWS, PAR-CLIP (Photo Activatable Ribonucleoside-enhanced CrossLinking and Immuno-Precipitation), a technique to study the RNA interactome of any RBP of interest [13], was previously applied to EWS revealing its transcriptome-wide RNA targets [14]. Using PAR-CLIP combined with stringent bioinformatic quality criteria we could show that the mRNAs of 4488 genes were directly bound by EWS. We now sought to further investigate the regulated among the many bound targets to uncover the physiological function of EWS. We further identified 116 genes whose expression altered upon EWS down regulation and show that a cell cycle regulator CCDC6 is regulated by EWS by binding to its 3’untranslated region (3’UTR).

## Material and Methods

### Cell lines and cell culture

For culture conditions of T-REx HEK293 Flp-In cells (Invitrogen), HEK293T cells and cells stably expressing FLAG/HA-tagged EWS please see [14]. Ewing sarcoma cell line MHH-ES-1 was purchased from DSMZ (Germany) [15–17] and grown in RPMI-1640 supplemented with 10% FBS. All of the above-mentioned cell lines were incubated at 37°C and 5% CO_2_.

### Plasmid constructs

For detailed descriptions of the EWS expression plasmids please refer to [14]. The EWS binding region sequences of the selected mRNA targets were cloned into pSI-CHECK2 vector (Promega) under the control of Renilla luciferase. Primer sequences are given in the S1 Table.

### Antibodies

Monoclonal anti-EWS (Abcam, AB54708), monoclonal anti-CCDC6 (Abcam, AB56353), monoclonal anti-ß actin antibody (Sigma Aldrich, A2228) were used as primary antibodies at 1:3000, 1:1000 and 1:10000 dilutions respectively. Secondary goat anti-mouse IgG-HRP antibody (Santa Cruz biotechnology, sc-2005) was used at 1:10000 dilution.

### siRNA transfections

HEK293T and MHH-ES-1 cell lines were reversely transfected with siRNAs using Lipofectamine RNAiMAX (Invitrogen). siRNAs targeting EWS were designed and obtained from Eurofins MWG operon. AllStars Negative Control siRNA was obtained from Qiagen. HEK293T and MHH-ES-1 cell lines were transfected with siRNAs at 50 nM concentration in 12 well plates for EWS, CCDC6 mRNA and protein analysis. 72h post transfection, cells were harvested for analysis. Transfection rates were in the range of 60–70% for MHH-ES-1 and 90% for HEK293T cell lines. For the sequence of the siRNA and the figure showing the region being targeted by the siRNA see the S1 Table and S1A Fig.

### Quantitative RT-PCR

Total RNA was extracted using the RNeasy Mini kit (Qiagen). cDNA was synthesized using Superscript Reverse Transcriptase Kit III (Invitrogen) according to the manufacturer’s instructions (oligo dT). Quantitative PCR was carried out using Power SYBR green kit (Applied Biosystems). All reactions were run on an ABI 7500 Real time PCR machine (Applied Biosystems) in triplicate. Data was acquired using the ABI SDS 2.0.1 software package. RNA isolated from the samples was tested for the expression levels of the chosen targets and their Δct values were subtracted from the respective beta actin expression levels.

### Western blot

Cells were harvested, resuspended in NP40 lysis buffer and lysed. A 10% SDS-PAGE gel was run in Tris-glycine-SDS buffer. A semi-dry transfer procedure was carried out onto cellulose membrane. After transfer, the membrane was blocked in TBS with Tween 20 and 5% milk. The membrane was probed with mouse monoclonal antibodies detecting CCDC6, EWS and beta actin. Horseradish peroxidase (HRP)-conjugated goat anti-mouse antibody was used as a secondary antibody. Chemiluminescence was used to detect EWS, CCDC6 and beta actin using the Super Signal West Pico chemiluminescent Substrate (Thermoscientific).

### Reporter assay

HEK293T cells were co-transfected with 25ng of psiCHECK-2 plasmid containing the target mRNA sequences and 50ng of EWS expression plasmid using Lipofectamine 2000 (Invitrogen) transfection reagent. 25ng of empty vector psiCHECK-2 along with 50ng of EWS expression plasmid was used as negative control. A total of 5x10^4^ cells were plated in 24 well plates 24h prior to transfection. 48h after transfection cell lysates were prepared according to the manufacturer’s instructions with the Dual luciferase assay system (Promega). Renilla and firefly luminescence was read using Luminoskan ascent microplate luminometer (Thermoscientific). Renilla/ firefly luciferase ratios were calculated from the mean values of triplicates. Data is represented as mean SEM (Standard error of mean) values.

### Cell cycle and cell death analysis using flow cytometry

Cells were prepared for cell cycle analysis and viability using propidium iodide staining of the DNA content. Cells were harvested by washing with PBS, trypsinized and pelleted via centrifugation at 1500 rpm for 5 minutes at RT and resuspended directly in the propidium iodide staining solution or Nicoletti buffer (0.1% sodium citrate, TritonX-100 and 50mg/ml of propidium iodide) The cells were incubated in the dark for 15 minutes at 4°C and about 50000 cells were analyzed on the BD FACScanto Flow cytometry machine (BD biosciences). To further distinguish apoptotic cells from necrotic cells, cells were stained with Annexin-V-FITC and counterstained with propidium iodide (PI). The cells were washed in cold PBS and resuspended in Annexin binding buffer at 1x 10^6^ cells per ml. 2 μl of Annexin-V-FITC (BD PharMingen) and 4 μl of PI (20 μg/ml) were added to 100 μl of cell suspension. The cells were incubated in the dark for 15 minutes at RT. After the incubation 100 μl of Annexin binding buffer was added and the cells were subsequently analyzed.

### CCK-8 assay to measure cell proliferation

Cell proliferation assay was performed using CCK-8 kit (Sigma-Aldrich) as per the manufacturer’s protocol. Cells were transfected with siRNAs against EWS as well as controls as previously described and seeded into 96 wells. Absorbance at 450nm was measured 4h after the addition of 10μl of CCK-8 reagent per well with 1*10^5^ cells. Readings were taken at 36, 60 and 84 hours using Tecan Photometer.

### mRNA stability assays

The mRNA half-life was determined by treating the cells with 3μg/μl Actinomycin D (ActD Sigma-Aldrich). Cells were collected at different time points (0–12) and total RNA was isolated followed by cDNA synthesis. Real time PCR, to measure the percentage of RNA remaining for EWS, CCDC6 along with ß-actin as an internal control was performed. A linear plot of the percentage RNA remaining and time of ActD treatment was plotted to calculate the mRNA decay constant.

### Statistical analysis

All experiments were performed at least in triplicates. Numerical data were expressed as mean ± SEMs. Group comparisons were analyzed by two way ANOVA or unpaired *t* test. P values were calculated and a p value of <0.05 was considered significant.

## Results and Discussion

### RNA-binding protein EWS binds and regulates the expression of 116 target mRNAs in HEK293T cells

In order to study the transcriptome of FET family of RNA binding proteins, we previously performed PAR-CLIP on all the three proteins and showed that mRNAs of 4488 genes were bound by EWS [14] To now identify the regulated among all bound mRNAs, we performed siRNA mediated knockdown experiments of EWS in HEK293T cells and analyzed mRNA expression changes using microarrays (S1B Fig.) In total, we found 116 (S2 Table) regulated genes with a corrected p-value of <0.05 (32 at <0.01)), which had more than one PAR-CLIP cluster and whose expression level changed (either up or down) by at least 50% upon knockdown of EWS compared to controls.

The regulated genes included tumor-associated genes such as *LIN28B* (regulates let-7 miRNA processing) [18], *PURB* (controls both DNA replication and transcription, its deletion has been associated with acute myeloid leukemia) [19], *CCDC6* (a potential tumor suppressor) [20], *CDCA4* (regulates E2F-dependent transcriptional activation and cell proliferation, mainly through the E2F/retinoblastoma pathway) [21], *MDM2* (E3 ubiquitin protein ligase and repressor of transcription factor p53) [22–24] and *FGF9* (roles in tissue repair, tumor growth and invasion) [25, 26]. Functional annotation using DAVID analysis [27, 28] showed that several genes are associated with diverse functions ranging from response to DNA damage, response to cellular stress, cell cycle process, translational initiation and cellular differentiation.

For our initial analysis we focused on the four highly regulated targets *CCDC6* (log fold change of-1.10), *MDM2* (-0.53), *FGF9* (-0.66) and *CBFB* (-0.58) which were all downregulated upon knockdown of EWS.

Analysis of the PAR-CLIP cluster binding localization revealed that these 116 regulated mRNAs were preferentially bound in the 3’UTR (60%) and had less intronic clusters compared to those of all bound 4488 mRNAs (40%). This effect was even more pronounced in the four chosen targets (75% 3’UTR binding) (Fig. 1B).

### EWS regulates a subset of genes with roles in several human cancers and in other cell survival mechanisms

The regulation of the four selected targets was further confirmed using qRT-PCR analysis. HEK293T cells which were treated with siRNA targeting EWS showed clear reduction in the expression of CBFB, CCDC6, MDM2 and FGF9 (Fig. 1C). Downregulation of these genes could further have an effect on genes they regulate and signaling pathways they are involved in. For example, MDM2 is a protein which inhibits activation of p53 [24], any downregulation in the levels of MDM2 disturbs the functional interactions of these networks which further affects tissue homeostasis [22] such as the notch induced p53 activity [29, 30]. Another target, FGF9, which was reported to be downregulated and mutated in several carcinomas plays an important role in activating FGFR signaling. This signaling pathway plays a role in differentiation and inhibits growth thus acting as a tumor suppressor. Therefore its downregulation upon EWS knockdown could repress its tumor suppressor activity [25, 26]. Similarly, CBFB is also frequently mutated in AML and is known to play a role in hematopoietic development [31]. The genes regulated by CBFB are important in chromatin deacetylation and promoter methylation and thus playing a role in gene transcriptional activation or repression. Therefore its downregulation could disrupt the normal pathways in hematopoiesis and also effect gene regulation. Future experiments are necessary to study in detail the interactions of EWS with these genes and the complex pathways they regulate.

Since ectopic expression of EWS/FLI1 resulted in growth arrest and apoptosis rather than promoting cellular transformation in cells [32] as well as in mice [33], we next sought to look at the genes regulated by EWS with roles in cellular proliferation, cell cycle and cell death. Several of the EWS up-regulated targets and down-regulated targets had roles in cell cycle and G1/S transition like SKP2 (p45), BCAT1, CCDC6, CUL2, PPP1CB, RHOU etc. Genes with roles in proliferation and cell death included DNAJA2, MDM2, NOP2, FGF9, ID2 etc. Others like AEN, APTX, TOP2A and UBE3E had roles in DNA damage response. We further narrowed down our focus onto CCDC6, Coiled-Coil Domain Containing 6 gene since it showed high fold change in the microarray analysis and a high number of PAR-CLIP clusters. CCDC6 is also known to have several cell cycle associated functions including DNA damage response [34, 35], cell cycle regulation by controlling the intra-S-Phase and G2/M checkpoints. Knockdown of CCDC6 also showed increased apoptosis and decreased proliferation [36, 37].

### CCDC6 is regulated by EWS on RNA and protein level

CCDC6 is a ubiquitously expressed protein which is downregulated in several cancers and predicted to be a tumor suppressor [20]. It is known to be frequently rearranged with RET protein in papillary thyroid carcinomas [38, 39] and fuses to platelet derived growth factor receptor beta gene causing atypical chronic myeloid leukemia [40].

RNA-binding proteins are known to regulate gene expression via post transcriptional mechanisms by stabilizing the mRNA they bind to. We wanted to explore whether EWS stabilizes CCDC6 by binding to it. We performed mRNA stability assay by treating the cells with Actinomycin D to inhibit de novo RNA synthesis and found that the half-life of CCDC6 was ~4.1 h in control cells, whereas in cells treated with EWS siRNA the half-life decreased to ~3.2 h. Together, these data suggest that EWS stabilizes CCDC6 transcript (Fig. 1D).

We also performed luciferase assays to confirm the regulation of CCDC6 mRNA by EWS protein. We cloned the mRNA target sequence carrying the PAR-CLIP clusters downstream of the 3’UTR of Renilla luciferase, and used firefly luciferase as an internal control in psiCHECK-2 plasmid. Upon co-transfection of both plasmids we observed an increase of luciferase activity compared to the empty vector control due to the increase in the expression of CCDC6 UTR (Fig. 1E). We then performed the luciferase assays with different doses of EWS and a clear dose-dependent effect was observed which further confirmed the specificity of EWS mediated regulation of CCDC6 (S1C Fig.).

**Fig 2:**
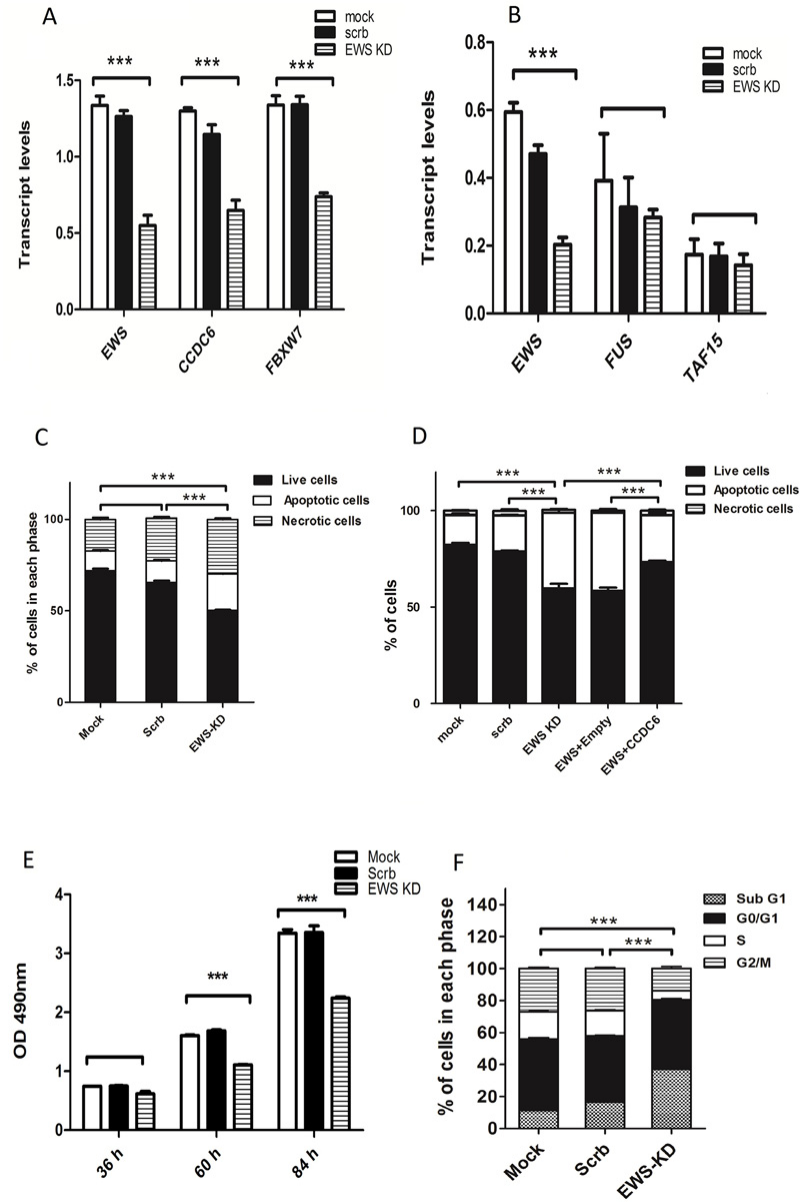
EWS downregulation affects apoptosis, cell cycle and proliferation. **A**. Decrease in the relative mRNA levels of FBXW7 upon knocking down EWS. **B**. Relative mRNA levels of FUS, EWS and TAF15 (FET family proteins) upon EWS knock down. **C**. Total percentage of living, necrotic and apoptotic cells after EWS and scrambled siRNA knockdown are represented on the bars. Apoptotic cells are defined by the sum of population of cells in early apoptosis and late apoptosis. Mock, scrambled and EWS KD had 10%, 11.8% and 20% apoptotic cells and 17%, 22% and 30% of necrotic cells respectively. The P values refer to the apoptotic cell population. **D**. Rescue effect upon co-transfection of EWS siRNA and CCDC6 overexpression. Bars represent total percentage of living, apoptotic and necrotic cells. 50nM siRNA, 100ng of empty vector and 100ng of CCDC6 expression vector were transfected. Mock and scrambled had 15% and 18.6% of apoptotic cells, EWS KD and EWS KD+ Empty vector had 39% and 40.5% apoptotic cells respectively and EWS KD+ CCDC6 vector had only 24% apoptotic cells. The P values refer to the apoptotic cell population. **E**. Proliferation rates on three consecutive days using CCK8 assay was calculated by measuring the absorbance which is proportional to the amount of living cells. **F**. Quantification of cell cycle distribution. The bars indicate the % of cells in each cell cycle phase subG1, G0/G1, S and G2/M phase. Mock has 11.3%, 44.7%, 16%, 28% of cells in subG1, G0/G1, S and G2/M phase respectively. 16%, 42%, 15%, 27% of cells for scrambled and 37%, 44%, 5%, 14% of cells for EWS KD in each phase respectively. The calculated P values refer to the cells in S phase. Data in all the above figures (A-F) is presented as mean SEM values (*P<0.05; where n = 3 per group).

For our further experiments, we chose MHH-ES-1, a Ewing sarcoma cell line which carries the most frequently occurring translocation EWS-FLI. qRT-PCR results showed downregulation of CCDC6 mRNA levels upon knockdown of EWS. The siRNA is designed to target the c-terminal region of EWSR1 which is absent in EWS-FLI1 fusion transcript in MHH-ES-1 (Fig. 1F). The same effect was also observed in HEK293T cells (S1D Fig.). Also, Western blot showed decreased expression of CCDC6 protein upon EWS knockdown in MHH-ES-1 (Fig. 1G) and also in HEK293T cells (S1D Fig.) thus showing that the regulation of CCDC6 by EWS extends to the protein level. Since this regulation might further have an impact on several other downstream genes that CCDC6 itself regulates, we tested one of these published targets, namely FBXW7, which is required for DNA damage response [41], and indeed, qRT-PCR on FBXW7 following knockdown of EWS showed reduced RNA levels (Fig. 2A). Since FBXW7 is mutated in several cancers and shown to be a tumor suppressor, the pathway through which all these proteins inter-regulate might further play a role in sarcomagenesis [42]. Further studies are required to elucidate the role of FBXW7 in Ewing sarcoma context.

Taken together, these results suggest that EWS regulates the expression levels of CCDC6 by stabilizing it and further exerting its effect on the expression of its downstream targets.

### No rescue effect by FET family members in Ewing sarcoma cell line

It was previously shown that FET proteins show redundancy and that knockdown of EWS upregulates its two family members FUS and TAF15 in HEK293 cells as well as in liposarcoma cell lines [43]. To address whether this mechanism might also lessen the effects of EWS haploinsufficiency in Ewing sarcoma, we measured FUS and TAF15 mRNA levels upon EWS knockdown in MHH-ES-1 cells. However, there was no increase in mRNA levels for either of the two genes (Fig. 2B). This indicates that, probably due to a different cellular context, in Ewing sarcoma there is no rescue mechanism for loss of EWS expression.

### Downregulation of EWS affects viability, proliferation and cell cycle

Gene set enrichment analysis and DAVID analysis revealed that several targets regulated by EWS play a role in regulating cell proliferation and cell cycle. Also, EWS was reported to maintain mitotic integrity and proneural cell survival in zebra fish by regulating aurora B [10]. Earlier studies reported that CCDC6 silencing increased apoptosis, decreased cell proliferation and affected cell cycle division by controlling the intra S phase and G2/M checkpoints [36]. TAF15, another member of the FET family of proteins was also shown to regulate cell proliferation and apoptosis *in vivo* [44].

To confirm whether downregulation of EWS also affects those processes, we assessed apoptosis using Annexin V FITC and PI double staining of EWS knockdown and control HEK293T cells. We recorded an increase in the percentage of apoptotic cells in EWS siRNA treated cells (63% living cells, 27% apoptotic and 10% necrotic cells) compared to controls (74% living, 13% apoptotic and 13% necrotic cells) (Fig. 2C) while the % necrotic cells remained unchanged. We also confirmed increased cell death using a trypan blue dye exclusion assay by counting the cell number of dead and vital cells on three consecutive days (data not shown). These results further confirmed the previous finding that EWS knockdown induces apoptosis [10]. We next performed gain of function experiments to see if up regulation of CCDC6 after EWS knockdown will rescue this phenotype. To do this, we co-transfected the cells with EWS siRNA and CCDC6 overexpression vector and found that the apoptotic rate were 14% less in CCDC6 overexpressed cells compared to empty vector (Fig. 2D), confirming that overexpression of CCDC6 upon EWS knockdown indeed rescued the observed phenotype.

Apoptotic signals are coupled to growth regulatory processes such as proliferation, cell cycle arrest, and cellular differentiation. Therefore to examine if the observed apoptosis affected the proliferation rate of the cells, we measured the proliferation of EWS knockdown and control cells using CCK8 assay. We indeed noticed that the proliferation rate was remarkably declined upon downregulation of EWS compared to those transfected with no siRNA transfection and scrambled siRNA transfection (Fig. 2E).

Given that EWS regulates the expression of CCDC6, and that EWS downregulation induces apoptosis, and CCDC6 has been implicated in apoptosis coupled to S and G2/M phase cell cycle defects, we next tested cell cycle progression of the cells by propidium iodide staining after 48 hours of transfection. As shown in Fig. 2F, we observed a decrease of cells in S and G2/M phase upon EWS knockdown and an increased cell number with a sub G0/G1 DNA content typical of apoptotic cells. This indicated increased apoptosis of cells that had passed the G1 checkpoint. Higher apoptotic rate might have likely affected the duration of cell cycle or the doubling time of the cells. The observed decrease of cell number in S and G2/M phase might be due to the fact that CCDC6 regulates cell cycle checkpoints like 14–3–3σ and CDC25C [37] which are important for S phase duration, transition into G2 phase and activation of mitosis. Cell cycle checkpoints safeguard the cells from accumulating genetic errors and any deregulation will prepare the ground for increased mutagenesis and onset of several cancers. Therefore the aberrant entrance of cells into next phases by skipping the checkpoint controls regulated by CCDC6 might further triggers unwanted mutations leading to genetic aberrations.

Taken together our results demonstrate that EWS controls apoptosis and cell cycle progression by regulating one of its key mRNA target CCDC6. EWS therefore regulates a wide range of cellular processes to ensure genome integrity and cellular homeostasis. Recent studies have unveiled new roles of EWS in gene regulation and RNA metabolism. This suggests that highly complex roles are played by EWS and a thorough elucidation of the entire mRNA target network of native EWS is essential in understanding the molecular and cellular biology of Ewing sarcoma. Future studies will show whether EWS and/or its target mRNAs will offer new therapeutic approaches.

## Supporting Information

S1 Fig **Location of siRNA on EWS (A)**. The arrow indicates the RRM region of EWSR1 targeted by siRNA thus exclusively targeting only the non translocated allele. **siRNA mediated knockdown of EWS for microarray analysis (B)**. Bars indicate transcript levels as measured using Affymetrix U133 Plus 2.0 arrays showing efficient EWS knockdown on mRNA level. Signal intensities were calculated by averaging redundant probe sets for the same gene. Error bars indicate standard error of mean (SEM). *: P < 0.001. ß actin was included for comparison. a.u., arbitrary units; ctrl., transfection of control siRNA. **Dose dependent regulation of CCDC6 by EWS in luciferase assay (C)**. Regulation of CCDC6 by EWS was further tested with increasing concentrations of EWS by measuring the relative luciferase activity. On the x axis the alphabets indicate the plasmids that were co transfected along with 50 ng psiCHECK-2-CCDC6 and the following co-plasmids accordingly. a) 25 ng of pDEST-EWS b) 50 ng of pDEST-EWS, c) 75 ng of pDEST-EWS d) 75 ng of empty pDEST e) 25 ng of empty pDEST f) 50 ng of empty pDEST g) 75 ng of empty pDEST h) 75 empty psiCHECK-2. **Downregulation of CCDC6 following EWS knockdown in HEK 293T cells (D)**. Relative mRNA levels of CCDC6 and EWS in wild type, control and EWS knockdown in HEK293T cells. Relative mRNA levels were normalized to beta actin. Western blot showing the downregulation of CCDC6 upon EWS knockdown in HEK293T cells. Antibodies are indicated.
(DOC)

S1 Table Sequences for primers and siRNA. (XLS)

S2 Table List of genes regulated by EWS. (XLS)
