# Supplementary material for: The Cell Cycle Regulator CCDC6 Is a Key Target of RNA-Binding Protein EWS
Source: PLoS One. 2015 Mar 9;10(3):e0119066. doi: 10.1371/journal.pone.0119066 (PMC4353705; doi:10.1371/journal.pone.0119066)

**S1.A Fig.**

**Location of siRNA on EWSR1.**

**
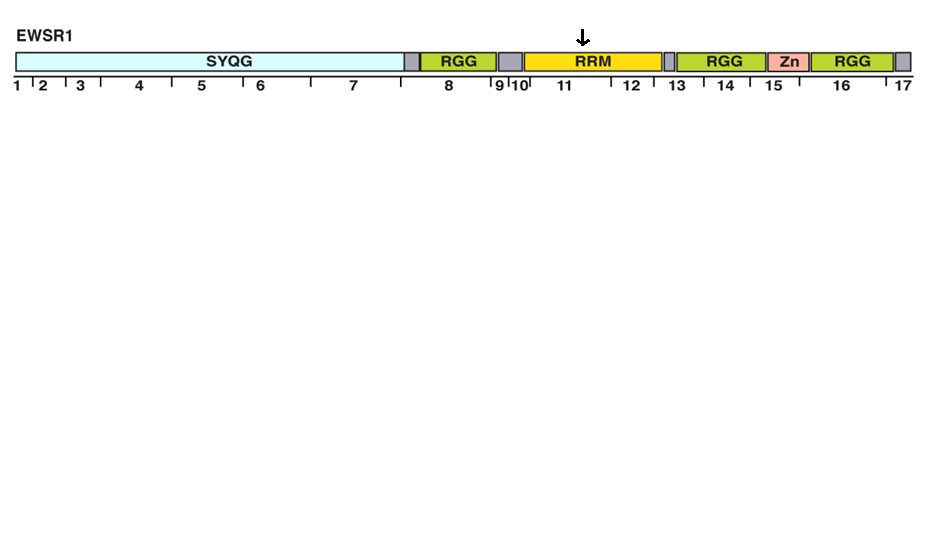
**

The arrow indicates the RRM region of EWSR1 targeted by siRNA thus exclusively targeting only the non translocated allele.

**S1.B Fig.**

**siRNA mediated knockdown of EWS for microarray analysis.**

Bars indicate transcript levels as measured using Affymetrix U133 Plus 2.0 arrays showing efficient EWS knockdown on mRNA level. Signal intensities were calculated by averaging redundant probe sets for the same gene. Error bars indicate standard error of mean (SEM). *: P < 0.001. ß actin was included for comparison. a.u., arbitrary units; ctrl., transfection of control siRNA.


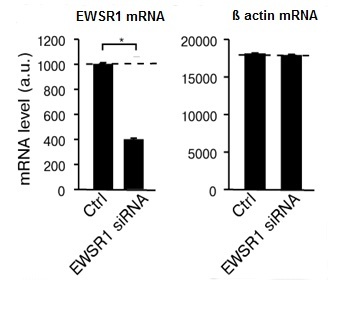


**S1.C Fig.**

**Dose dependent regulation of CCDC6 by EWS in luciferase assay.**

Regulation of CCDC6 by EWS was further tested with increasing concentrations of EWS by measuring the relative luciferase activity. On the x axis the alphabets indicate the plasmids that were co transfected along with 50 ng psiCHECK-2-CCDC6 and the following co-plasmids accordingly. a) 25 ng of pDEST-EWS b) 50 ng of pDEST-EWS, c) 75 ng of pDEST-EWS d) 75 ng of empty pDEST e) 25 ng of empty pDEST f) 50 ng of empty pDEST g) 75 ng of empty pDEST h) 75 empty psiCHECK-2.


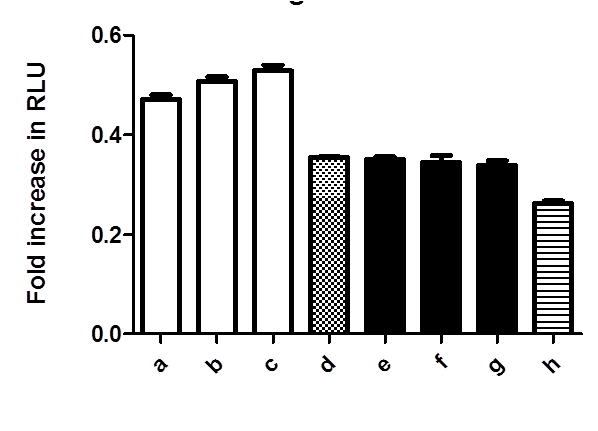


**S1.D Fig.**

**Downregulation of CCDC6 following EWS knockdown in HEK 293T cells.**

A) Relative mRNA levels of CCDC6 and EWS in wild type, control and EWS knockdown in HEK293T cells. Relative mRNA levels were normalized to beta actin.

B) Western blot showing the downregulation of CCDC6 upon EWS knockdown in HEK293T cells. Antibodies are indicated.


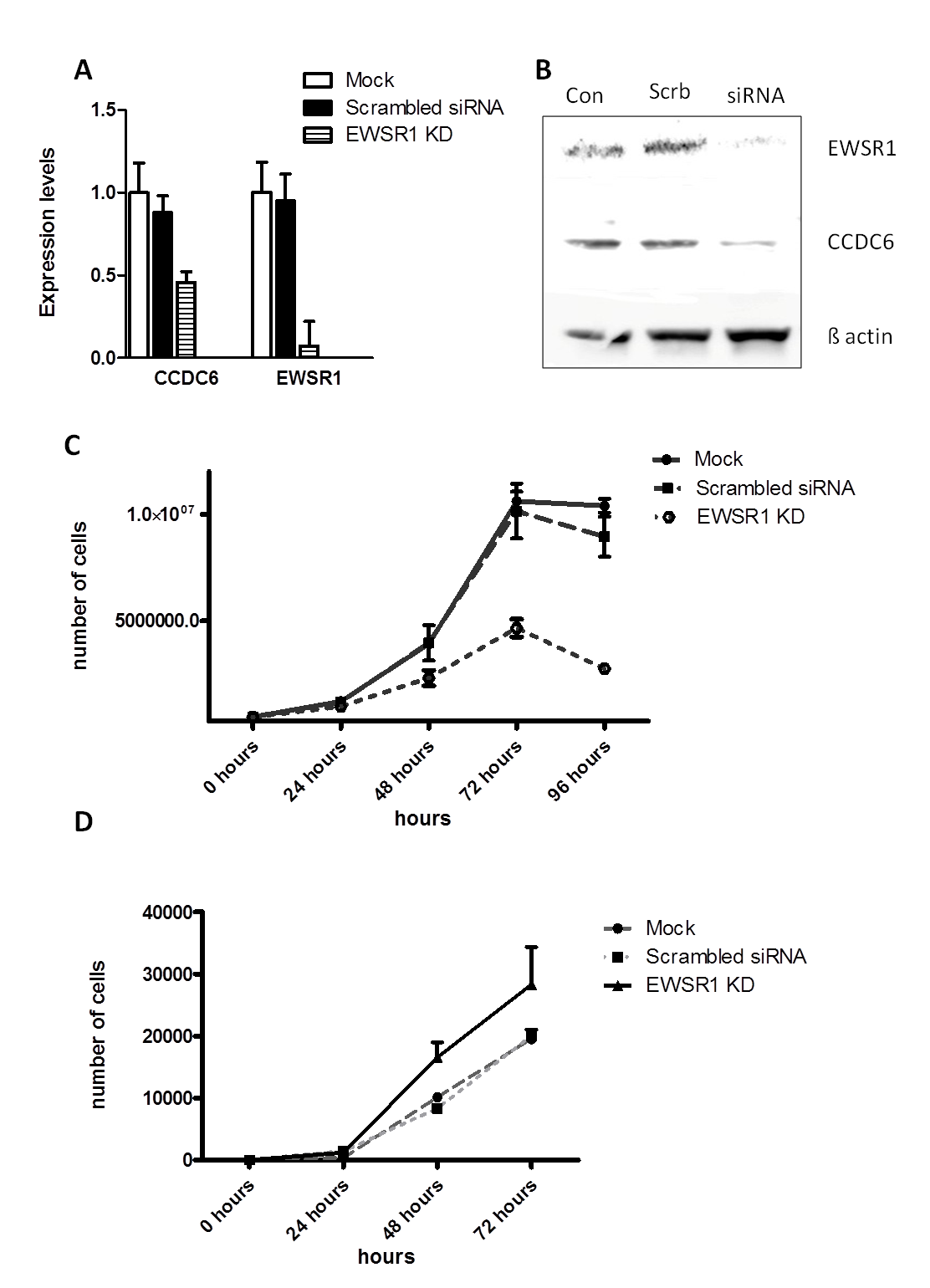

Supplement: S1 Fig — Location of siRNA on EWS (A). The arrow indicates the RRM region of EWSR1 targeted by siRNA thus exclusively targeting only the non translocated allele. siRNA mediated knockdown of EWS for microarray analysis (B). Bars indicate transcript levels as measured using Affymetrix U133 Plus 2.0 arrays showing efficient EWS knockdown on mRNA level. Signal intensities were calculated by averaging redundant probe sets for the same gene. Error bars indicate standard error of mean (SEM). *: P < 0.001. ß actin was included for comparison. a.u., arbitrary units; ctrl., transfection of control siRNA. Dose dependent regulation of CCDC6 by EWS in luciferase assay (C). Regulation of CCDC6 by EWS was further tested with increasing concentrations of EWS by measuring the relative luciferase activity. On the x axis the alphabets indicate the plasmids that were co transfected along with 50 ng psiCHECK-2-CCDC6 and the following co-plasmids accordingly. a) 25 ng of pDEST-EWS b) 50 ng of pDEST-EWS, c) 75 ng of pDEST-EWS d) 75 ng of empty pDEST e) 25 ng of empty pDEST f) 50 ng of empty pDEST g) 75 ng of empty pDEST h) 75 empty psiCHECK-2. Downregulation of CCDC6 following EWS knockdown in HEK 293T cells (D). Relative mRNA levels of CCDC6 and EWS in wild type, control and EWS knockdown in HEK293T cells. Relative mRNA levels were normalized to beta actin. Western blot showing the downregulation of CCDC6 upon EWS knockdown in HEK293T cells. Antibodies are indicated. (DOC) [file pone.0119066.s001.Doc]
